# Limited vertical transmission of microbiomes through the chorioallantoic membrane affects intestine development and metabolic pathways in broiler embryos

**DOI:** 10.3389/fmicb.2026.1780310

**Published:** 2026-07-13

**Authors:** Sadid Al Amaz, Suman Poudel, Md Ahosanul Haque Shahid, Rajesh Jha, Birendra Mishra

**Affiliations:** Department of Human Nutrition, Food and Animal Sciences, College of Tropical Agriculture and Human Resilience, University of Hawaiʻi at Manoa, Honolulu, HI, United States

**Keywords:** Gene expression, gut microbiome, immunity, incubation, microbiome profiling

## Abstract

**Introduction:**

The chorioallantoic membrane (CAM) in chickens is a vascularized structure that plays a crucial role in embryonic growth and development by facilitating gas exchange, nutrient transport, and waste removal. Understanding the microbial diversity and immune status of the CAM can provide valuable insights into the gut microbiome and post-hatch growth. Embryonic thermal manipulation (TM) represents an effective approach for enhancing broiler production. This study investigated the effects of TM on CAM and intestinal microbiota composition, metabolic pathways, and immunity-related gene markers.

**Methods:**

For embryonic TM, fertile Cobb 500 eggs (*n* = 600) were incubated in three incubators at a standard temperature of 37.5 °C with 55% relative humidity (RH) for the first 11 embryonic days (ED). After candling, on ED 12, eggs were allocated to two groups: (1) Control (*n* = 236) maintained at 37.5 °C and 55% RH for 24 h/d until hatch day (ED 21), and (2) TM group (*n* = 238) subjected to 38.5 °C and 55% RH for 12 h/d from ED 12 to ED 18, followed by standard temperature from ED 19 to ED 21 in two incubators with automatic temperature control, 55% RH.

**Results:**

Two newly identified bacterial genera, *Phenylobacterium* and *Limnobacter,* were found in CAM. Three previously unreported bacterial genera, *Phyllobacterium, Flavobacterium,* and *Rubelimicrobium,* were found in the intestine. The bacterial genera *Achromobacter, Stenotrophomonas, Lactobacillus, and Staphylococcus* showed the highest overlap in the CAM and intestines. TM significantly increased (*p* < 0.05) CAM and intestines’ microbial diversity (alpha and beta diversity) and metabolic microbial pathways. The mRNA expressions of *IL1B, IL10, IL12*, and *IL18* were significantly higher (*p* < 0.05) in the D18TM compared to the other treatment groups in the CAM.

**Conclusion:**

The study provides initial evidence of limited vertical transmission of microbiomes through the CAM into the intestine, along with characterization of CAM microbiomes. Embryonic TM significantly influenced both the diversity of CAM and intestinal microbiota, their associated metabolic pathways, and CAM immunity.

## 1 Introduction

The chorioallantoic membrane (CAM) serves as the respiratory organ for avian embryos (Nowak-Sliwinska et al., 2014). Embryonic development in commercial broilers (e.g., Cobb 500) lasts about 21 days under standard incubation conditions. The chorion and allantois fuse to form the CAM within 4 to 5 d of incubation (Hamburger and Hamilton, 1992). CAM is a simple yet highly vascular extraembryonic membrane that serves various functions during embryonic development, including, but not limited to, gas exchange (Nowak-Sliwinska et al., 2014). While the vascular system receives the majority of focus, it is crucial to highlight that the CAM possesses a fully developed lymphatic system that exhibits significant functional and molecular similarities to mammalian lymphatics (Papoutsi et al., 2001). The CAM model has been effectively utilized across various disciplines, including biology, medicine, and bioengineering, to investigate hemodynamics, immune cell migration, transplantation, and therapeutic responses, thereby ascertaining vascular responses and examining blood vessel and lymphatic morphogenesis and physiology.

The gastrointestinal (GI) tract is a crucial organ system responsible for nutrient absorption. During embryogenesis, molecular signals and mechanical forces shape the gut through intricate morphogenetic events culminating in its adult form. The chicken intestine develops from the embryonic endoderm’s anterior and posterior intestinal openings approximately at embryonic day (ED) 3 (Huycke and Tabin, 2018). Intestinal peristaltic waves commence approximately at ED 5 (Chevalier et al., 2017). At this stage, the gut is a simple composite tube that is suspended from the abdominal wall by the dorsal mesentery and made up of an endodermal epithelium covered in mesenchyme that was brought in from the splanchnic section of the lateral plate mesoderm. Shortly after its formation, the gut is populated anterior to posterior by neural crest cells originating from the ectoderm. The development and function of the digestive tract result from molecular and mechanical interactions among all three germ layers (Huycke and Tabin, 2018). Starting from ED 14, various levels of functional differentiation, morphological changes, and molecular alterations occur. Nonetheless, the intestinal capacity is enhanced when the chicken embryo ingests amniotic fluid around ED 17. At ED 17, both small and large villi are present; however, by ED 20, large villi constitute 70% of the total villi (El Sabry and Yalcin, 2023). The gut harbors a complex microbial ecosystem, including trillions of commensal microorganisms that coexist symbiotically with the host. The interactions between the host and the GI microbiome are essential for chickens’ physiological development, health, nutrition, and food safety (Oakley et al., 2014). The chicken GI tract microbiome is particularly vulnerable to interventions during early life, as supported by the competitive exclusion principle, which proposes that infections are less effective at colonizing the gastrointestinal tract when the native microbiome is more diverse later in life (Jurburg et al., 2019). They always maintain a complex bidirectional interaction with nutrition and overall immune condition (Shahid et al., 2026a). Throughout embryonic development, environmental variables and host genetic variation affected the diversity and abundance of the gut microbiota (Ding et al., 2017). These findings endorse the idea that the avian embryo may encounter microbial signals during development, potentially affecting host physiological and immunological maturation. Thermal manipulation (TM) could serve as an additional environmental factor influencing embryonic physiology, encompassing metabolic and immunological responses that may affect microbial community structure and host–microbe interactions.

**Figure 1:**
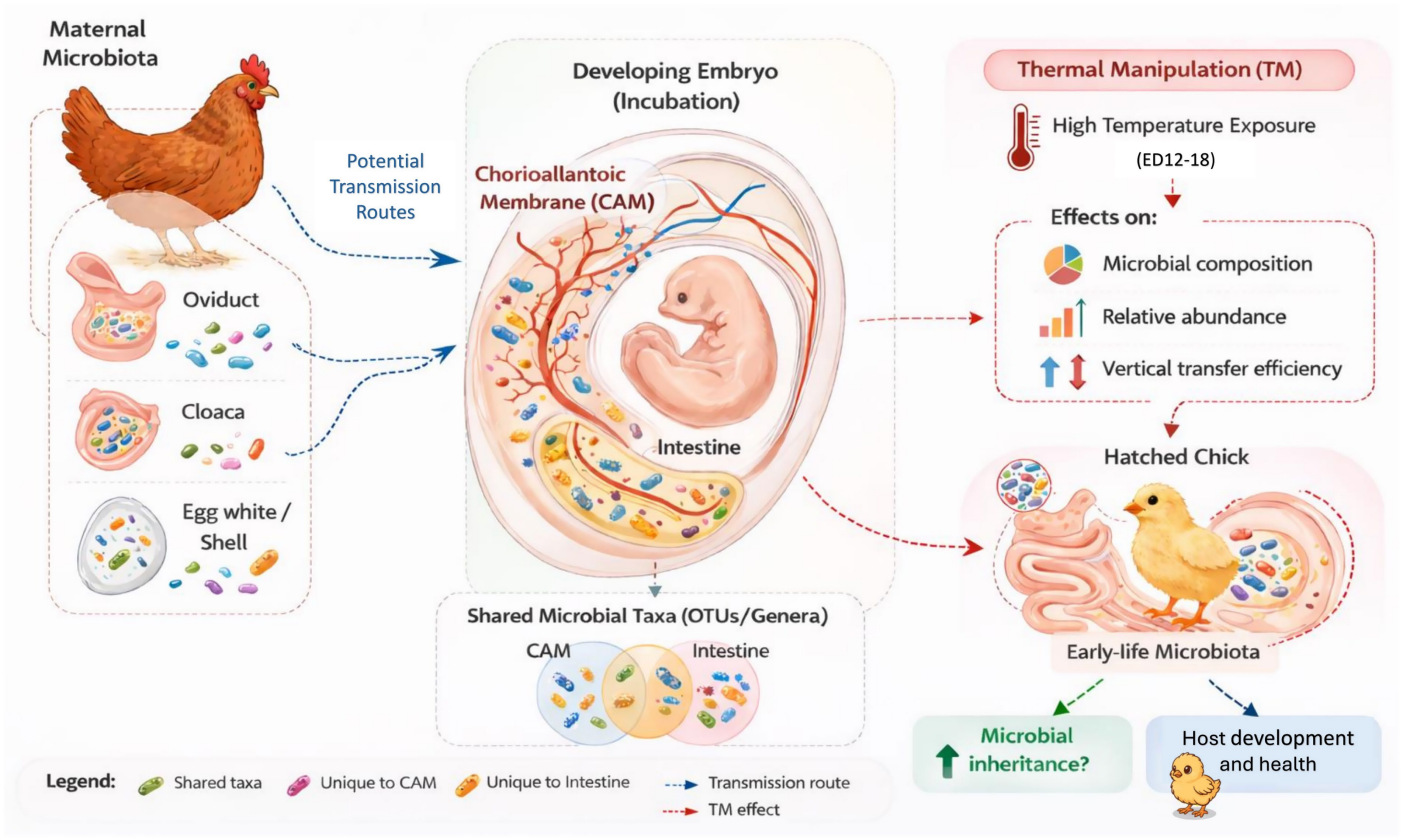
Conceptual model illustrating potential vertical microbial transmission routes and the effects of embryonic thermal manipulation (TM) during incubation (initially made by AI, later modified).

Thermal manipulation is a program of early heat exposure during embryonic development that has been suggested to enhance avian thermal tolerance and welfare without impacting post-hatch development (Al Amaz and Mishra, 2024). Our prior research indicated that embryonic TM improved hatchability, thermotolerance, liver metabolism, while decreasing hatch duration (Amaz et al., 2024) and early immunity (Amaz et al., 2025b). Furthermore, prehatch TM and post-hatch baicalein supplementation improved body weight, average daily gain, average daily feed intake, feed conversion ratio, cecal microbial diversity, and volatile fatty acids concentration (Al Amaz et al., 2024a, 2025b), liver metabolism, muscle cell proliferation (Al Amaz et al., 2024b), and immunity (Amaz et al., 2025a) in heat-stressed broilers. Based on the efficacy of embryonic TM, we hypothesized that embryonic TM would enhance the composition, pathways, and immunity of the CAM and intestinal microbiota. This study aims to (1) investigate the microbial diversity, metabolic pathways, and immune status present in the CAM and the intestine, and (2) examine the effects of embryonic TM on microbial diversity, metabolic pathways, and immune status within the CAM and the intestine. A conceptual model illustrating potential vertical microbial transmission routes and the effects of TM during incubation is shown in Figure 1.

## 2 Methods

### 2.1 Experimental design

The Institutional Animal Care and Use Committee (IACUC) at the University of Hawaii approved all animal research protocols (Approval No. 17-2605-6). For embryonic TM, Fertile Cobb 500 eggs (*n* = 600) were obtained from a local hatchery (Asagi Hatchery Inc., Honolulu, HI). All eggs were incubated in three incubators (GQF incubator, Savannah, GA; 200 eggs each) at a standard temperature of 37.5 °C with 55% relative humidity (RH) continuously for the first ED 11. Candling was performed on ED 10 to isolate the live embryos (*n* = 474). On ED 12, eggs were divided into two groups: (1) Control group (*n* = 236), which was maintained at 37.5 °C and 55% RH for 24 h a day until hatch day (ED 21), and (2) TM group (*n* = 238), which was subjected to 38.5 °C and 55% RH for 12 h/d from ED 12 to ED 18. From ED 19 to ED 21, the TM group was kept at standard temperature (37.5 °C). Both groups were incubated in separate incubators equipped with automatic temperature control and RH management, along with egg rotation every 2 h. Eggs from each treatment group were randomly assigned to incubators that maintained consistent temperature, humidity, and ventilation settings to eliminate any potential batch effects related to the incubators. No measurements were taken of the eggs’ or incubators’ temperatures, but the temperature did rise as set by the incubator. The hatching rate for the control group was 91%, while the treatment group (TM) was 94.5% (Amaz et al., 2024).

### 2.2 Sample collection

CAM and whole-intestine tissue samples were obtained from the Control and TM groups on ED 15 and ED 18 (*n* = 6) before initiating the TM. As the TM started at 11 a.m., we collected samples before 11 a.m. on each sampling day. We partially followed a previous protocol (De Goffau et al., 2019) because no established protocol existed for collecting CAM tissue in chickens. We collected 10–12 mm of CAM tissue using a single-use sterile scalpel and forceps to minimize contamination. The tissue in the chosen regions showed no signs of infarction, hematoma, or injury. The selected tissue samples were washed in cooled sterile PBS prepared with ultrapure water to eliminate blood. The complete experimental method, including tissue cutting, DNA separation, and PCR reaction setup, was conducted within a biological safety cabinet to mitigate the potential of environmental contamination of the materials. This study did not involve the incubation of specific pathogen-free fertile eggs, nor were samples collected from eggshells, incubators, or the surrounding environment. This approach aligns with standard practices within the poultry industry, where such procedures are not commonly undertaken. We collected the entire intestine because distinguishing its different parts is difficult during ED 15–18. The egg was initially broken into a sterile Petri dish, followed by the euthanization of the embryos through carbon dioxide asphyxiation for sampling purposes. No disinfected was used on the eggshell surface prior to cracking. Embryos were euthanized via carbon dioxide (CO₂) inhalation in accordance with AVMA recommendations, utilizing a gradual-fill approach (about 20–30% chamber volume displacement per minute) until loss of consciousness, followed by sustained exposure for a minimum of 2 min to sacrifice. The CAM and whole intestine (intestinal contents were not removed) were collected, rapidly frozen, and preserved at −80 °C until DNA and RNA extraction.

### 2.3 DNA extraction and 16S rRNA gene sequencing

As described previously (Al Amaz et al., 2024a), DNA was extracted, the 16S rRNA gene was sequenced, and the total genomic DNA was extracted from CAM and intestine using QIAamp® DNA Blood and tissue Kit and stool mini kit (Qiagen, Hilden, Germany) according to the manufacturer’s instructions, respectively. The V3–V4 region of the 16S rRNA gene was amplified using the universal primers 341F (5′-CCTACGGGNGGCWGCAG-3′) and 805R (5′-GACTACHVGGGTATCTAATCC-3′). NanoDrop One (Thermo Fisher Scientific, Madison, WI) measured bacterial DNA concentration and viability. Following the Illumina 16S Metagenomic Sequencing Library protocol (Illumina), the V3-V4 hypervariable regions of the 16S rRNA gene were amplified with the following change. The PCR reaction used Platinum Taq DNA Polymerase High Fidelity (Invitrogen, Life Technologies Corporation, Grand Island, NY), Mag-Bind Total Pure NGS beads (Omega Bio-Tek, Norcross, GA) for clean-up, and 35 cycles. Finally, Illumina MiSeq sequenced, normalized, and aggregated amplicons.

### 2.4 DNA sequence analysis

The CLC Microbial Genomics module and Genomics Workbench 25.0 were used for microorganism bioinformatics analysis. Sequencing analysis followed the operational taxonomical units (OTUs) clustering step-by-step tutorial (Qiagen, Hildesheim, Germany). Sequencing CAM samples generated 1,904,513 paired-end reads (mean: 63,483 reads per sample; range: 47,422–83,599). After quality filtering, 1,435,418 high-quality reads were retained and clustered into 1,225 OTUs. Sequencing of intestinal samples produced 1,156,364 paired-end reads (mean: 38,545 reads per sample; range: 30,935 - 48,469), of which 447,848 high-quality reads remained and were clustered into 549 OTUs. Rarefaction curves approached saturation at approximately 10,000–15,000 reads per sample, indicating that sequencing depth was sufficient for downstream analyses. As described (Al Amaz et al., 2024a), the CLC workbench paired, trimmed, and filtered the fastq files of demultiplexed sequences to remove low-coverage reads. Based on 97% sequence similarity to the Greengenes v13_8 97% database, the CLC Microbial Genomics module grouped filtered reads into OTUs. The phylogenetic tree was constructed using a maximum-likelihood method based on MSA of MUSCLE-generated OTU sequences in the workbench for alpha- and beta-diversity analyses. A boxplot showed Simpson’s index and Shannon entropy estimates of alpha diversity. A principal coordinate analysis (PCA) display of unweighted and weighted UniFrac distances estimated beta diversity. PERMANOVA was used to assess beta diversity. After eliminating OTUs with an abundance of less than 10, one-way ANOVA on the OTU table determined differentially abundant taxa (order, family, and genus), and Fisher’s Least Significant Difference test separated treatment groups by means.

### 2.5 Microbial metabolic pathway analysis

The functional metabolic pathways of cecal microbes were analyzed using QIIME2 platform. Filtered sequences obtained during microbiome characterization, along with the MetaCyc pathway database, were used to predict metabolic pathways. Phylogenetic Investigation of Communities by Reconstruction of Unobserved States (PICRUSt2) was used to infer functional traits from bacterial abundance. NSTI values generated by PICRUSt2 were used to evaluate the reliability of functional predictions. In both datasets, NSTI distributions were right-skewed due to a small number of OTUs with elevated values. For the CAM samples, NSTI values were generally low (median = 0.0187; interquartile range: 0.0080–0.0529), with approximately 86% of OTUs ≤ 0.15 and only ~2.9% exceeding 1, indicating close phylogenetic proximity to reference genomes and high prediction accuracy. In contrast, the intestine samples exhibited moderately higher NSTI values (median = 0.0434; interquartile range: 0.0219–0.1543), with ~74.8% of OTUs ≤ 0.15 and ~4.4% exceeding 1, suggesting a greater proportion of taxa with limited reference genome representation. To minimize the influence of extreme values, OTUs with NSTI > 1 were excluded, resulting in mean NSTI values of 0.062 ± 0.13 for CAM and 0.104 ± 0.158 for intestine samples. Collectively, these results indicate that functional predictions were robust for both datasets, with higher confidence in CAM samples and acceptable reliability in intestine samples. White’s non-parametric two-sided t-test was used to analyze taxonomic and functional profiles (STAMP v2) with a DP bootstrap at the 0.95 CI (Shahid et al., 2026b).

### 2.6 Quantitative real-time PCR (qPCR)

Total RNA was extracted from CAM and the intestinal tissue. RNA concentration was quantified using a NanoDrop™ spectrophotometer (Thermo Fisher Scientific, Madison, WI). Then, transcribed into cDNA and examined via qPCR following the established protocol (Al Amaz et al., 2025a). The primer sequences utilized for gene expression analysis are enumerated in Supplementary Table 1. Post-amplification, cycle threshold (Ct) values were documented, and gene expression levels were determined using beta-actin (*β-actin*) as the reference gene, with the 2 ^-ΔΔCt^ method. The stability of *β-actin* expression was evaluated prior to relative quantification by analyzing the consistency of Ct values among samples within each tissue dataset. In the CAM samples, *β-actin* Ct values ranged from 13.322 to 18.840, exhibiting a coefficient of variation (CV) of 7.63%. Meanwhile, Ct values in intestinal samples ranged from 13.303 to 16.046, exhibiting a CV of 4.84%. The results confirmed that *β-actin* is a valid reference gene for normalization in the 2^-ΔΔCt^ method.

### 2.7 Statistical analysis

The CLC Microbial Genomics module employed the Kruskal–Wallis pairwise test for alpha diversity and the PERMANOVA test for beta diversity. Microbial abundances and metabolic pathways were examined in STAMP 2.1.3 utilizing a two-sided White’s nonparametric t-test with a bootstrap parameter of 0.95. The Benjamini–Hochberg FDR adjustment was used to adjust *p*-values from differential abundance studies to account for false positives arising from multiple comparisons. Gene expression was evaluated using GraphPad (GraphPad Software, San Diego, CA). After conducting a two-way analysis of variance (ANOVA), the Tukey-HSD test was employed to compare the means of the different treatment groups. All data are expressed as mean ± SEM. The threshold for statistical significance was established at *p* < 0.05.

## 3 Result

### 3.1 CAM microbiota profile

**Figure 2:**
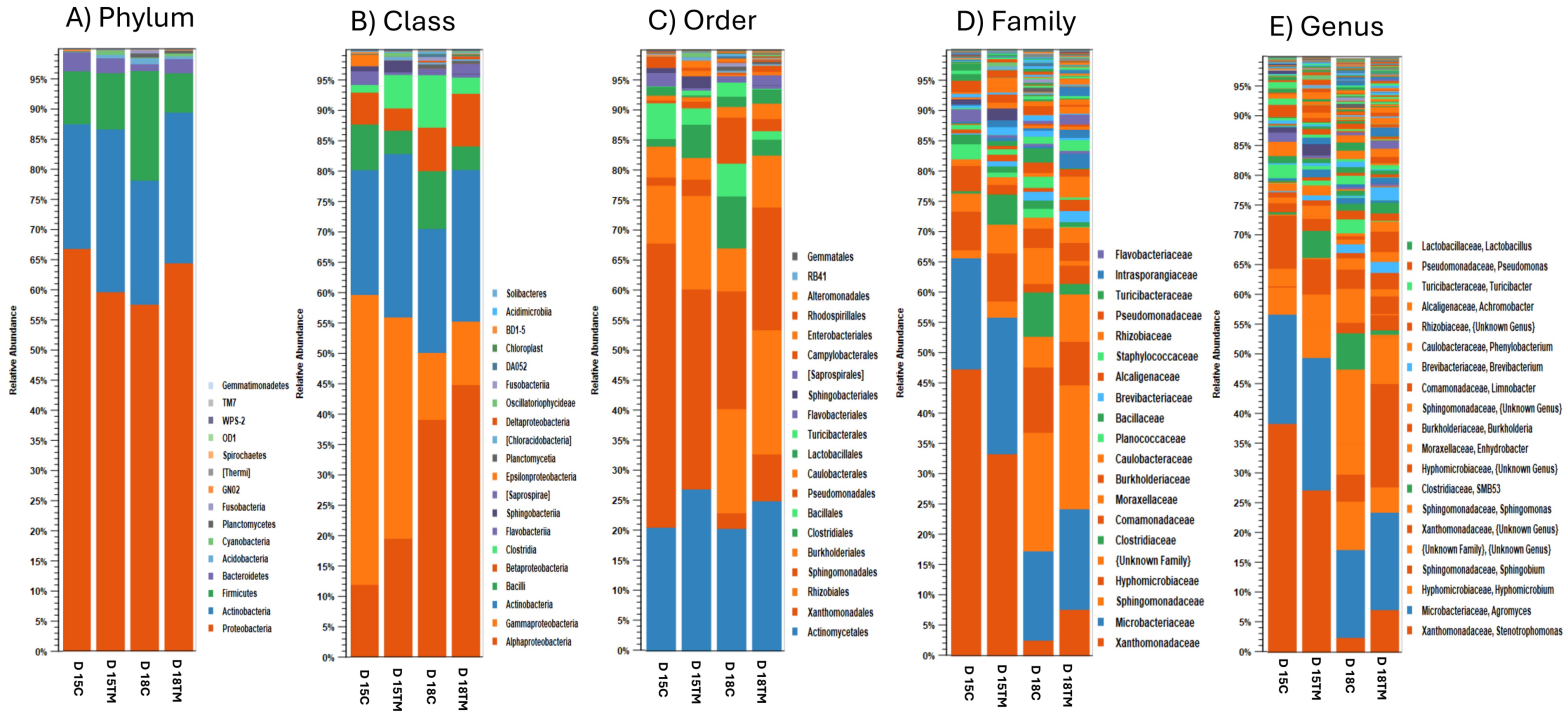
Effects of TM on the average relative abundance of the CAM microbiota at the phylum **(A)**, class **(B)**, Order **(C)**, Family **(D)**, and Genus levels **(E)**. Data represents relative abundance (%).

This study investigated CAM microbial profiling at the phylum, class, order, family, and genus levels. The diversity of CAM microbiota at the phylum level among treatment groups is shown after excluding low-abundance OTUs (Figure 2A). *Proteobacteria* and *Actinobacteria* were the two most dominant phyla among the treatments in D15C (67 and 20%, respectively), D15TM (59 and 30%, respectively), D18C (57 and 20%, respectively), and D18TM (65 and 25%, respectively).

At the class level (Figure 2B), the most prevalent microbiota were *Alphaproteobacteria* and *Gammaproteobacteria*. They were present in D15C (12 and 48%, respectively), D15TM (20 and 36%, respectively), D18C (39 and 11%, respectively), and D18TM (45 and 11%, respectively).

At the order level (Figure 2C), the most dominant microbiota were *Actinomycelates, Xanthomonadales,* and *Rhizobiales*. They were present in D15C (21, 47, and 8%, respectively), D15TM (26, 34, and 25%, respectively), D18C (20, 2, and 19%, respectively), and D18TM (25, 8, and 21%, respectively).

At the family level (Figure 2D), the most dominant microbiota were *Xanthomonadaceae*, *Microbacteriaceae*, and *Sphignomonadaceae*. They were present in D15C (47, 21, and 2%, respectively), D15TM (33, 23, and 3%, respectively), D18C (2, 15, and 20%, respectively), and D18TM (7, 17 and 21%, respectively).

At the genus level (Figure 2E), the most dominant microbiota were *Stenotrophomonas, Agromyces, Hyphomicrobium*, and *Sphingobium*. They were present in D15C (38, 19, 5, and 0.5%, respectively), D15TM (27, 22, 11, and 6%, respectively), D18C (3, 14, 9 and 17%, respectively), and D18TM (7, 12, 4 and 17%, respectively).

### 3.2 Alpha and beta diversity of CAM microbiota

**Figure 3:**
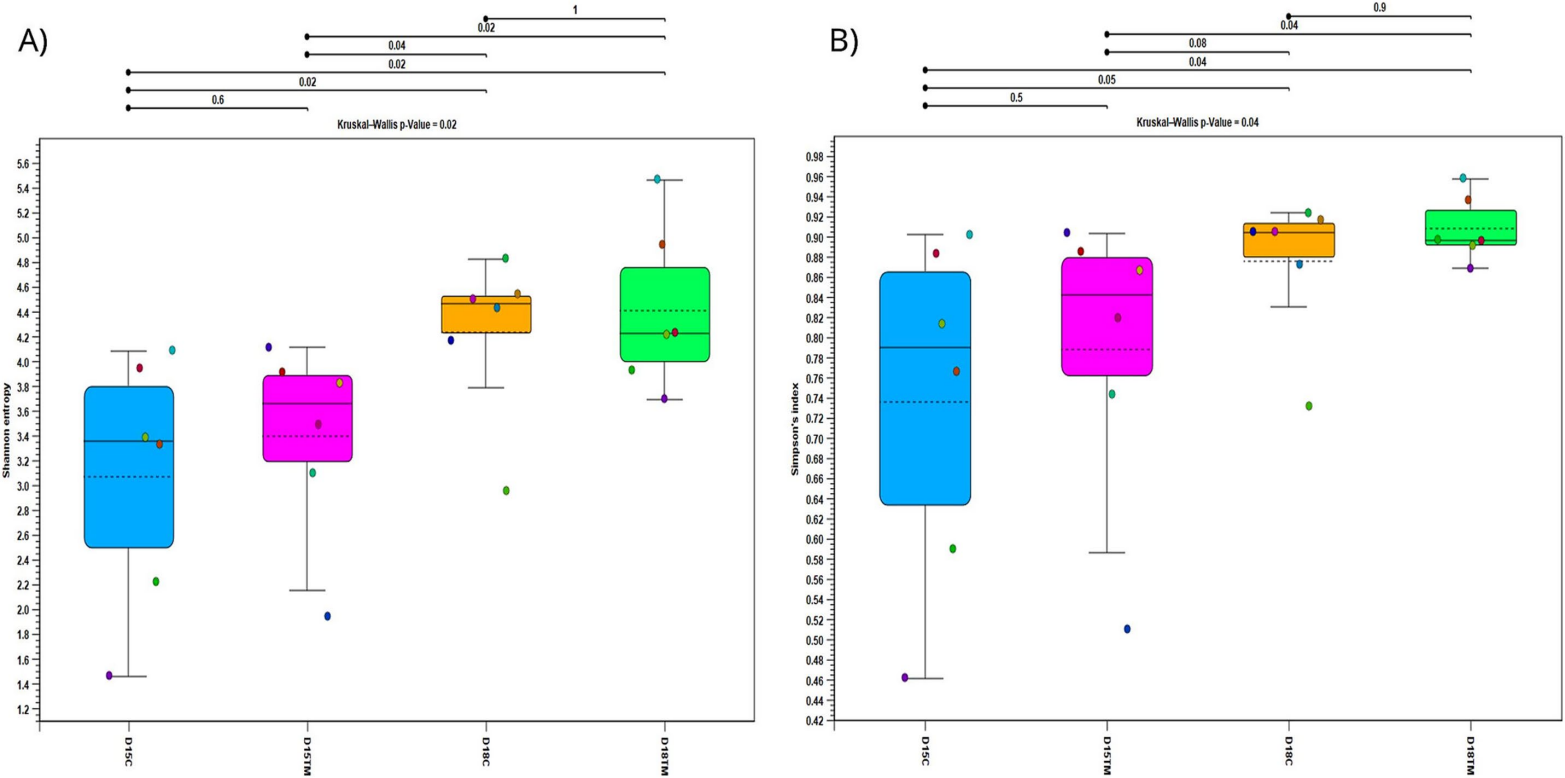
Effects of TM on microbial alpha diversity. **(A)** Shannon entropy. **(B)** Simpson’s index. The effects of treatments were significantly different at *p* < 0.05 among the treatment groups.

This study employed Shannon entropy and Simpson’s index to assess Alpha diversity (Figure 3). In this study, Shannon entropy showed a significant difference (*p* < 0.05) in the D18C and D18TM compared to D15C and D15TM. Simpson’s index was significantly different (*p* < 0.05) in the D18C compared to D15C. The D18TM was significantly different (*p* < 0.05) compared to D15C and D15TM.

**Figure 4:**
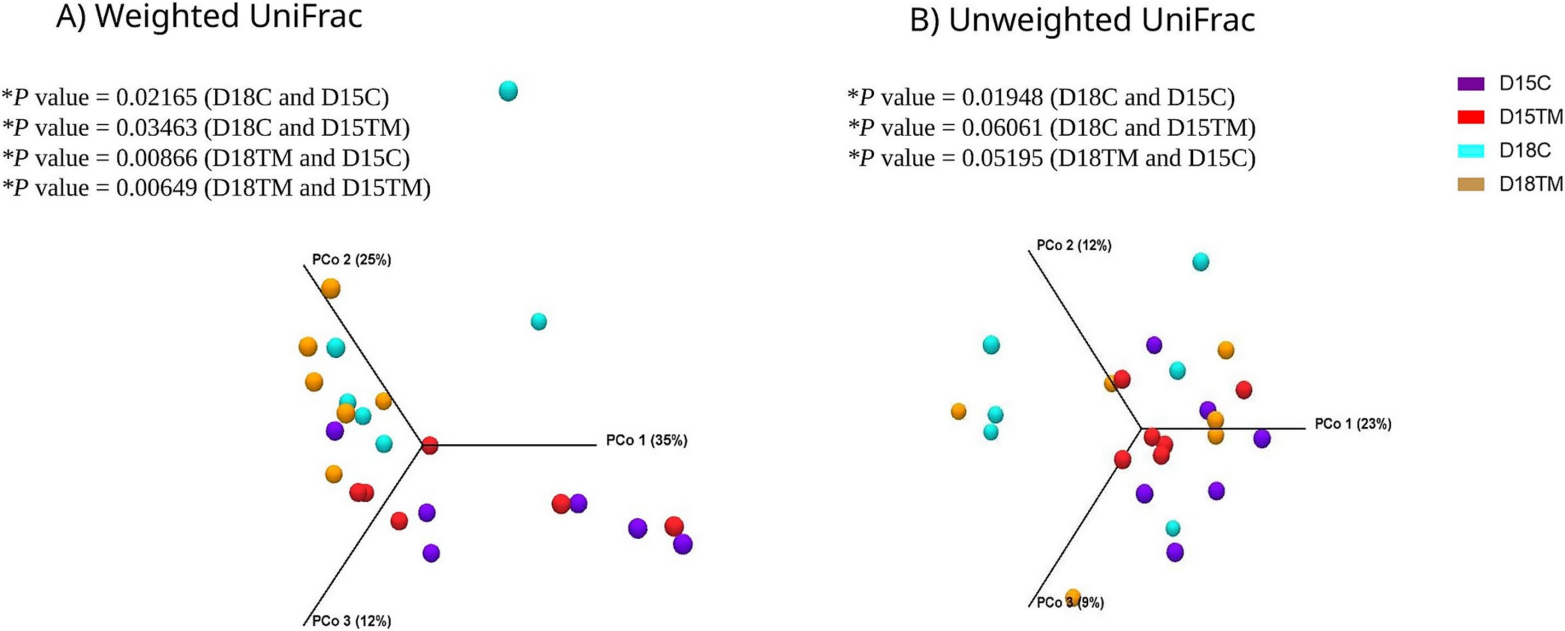
Effects of TM on microbial beta diversity. **(A)** Weighted UniFrac; **(B)** Unweighted UniFrac. The effects of treatments were significantly different at *p* < 0.05 among the treatment groups.

This study assessed Beta diversity through weighted UniFrac and unweighted UniFrac (Figure 4). The weighted UniFrac shows a significant difference in the D18C group compared to the D15C and D15TM (*p* = 0.02165 and *p* = 0.03463, respectively) and the D18TM group compared to the D15C and D15TM (*p* = 0.00866 and *p* = 0.00649, respectively). The Unweighted UniFrac shows a significant difference in the D18C group compared to the D15C and D15TM (*p* = 0.01948 and *p* = 0.06061, respectively), and the D18TM group compared to the D15C (*p* = 0.05195).

### 3.3 CAM microbial metabolic pathways

**Figure 5:**
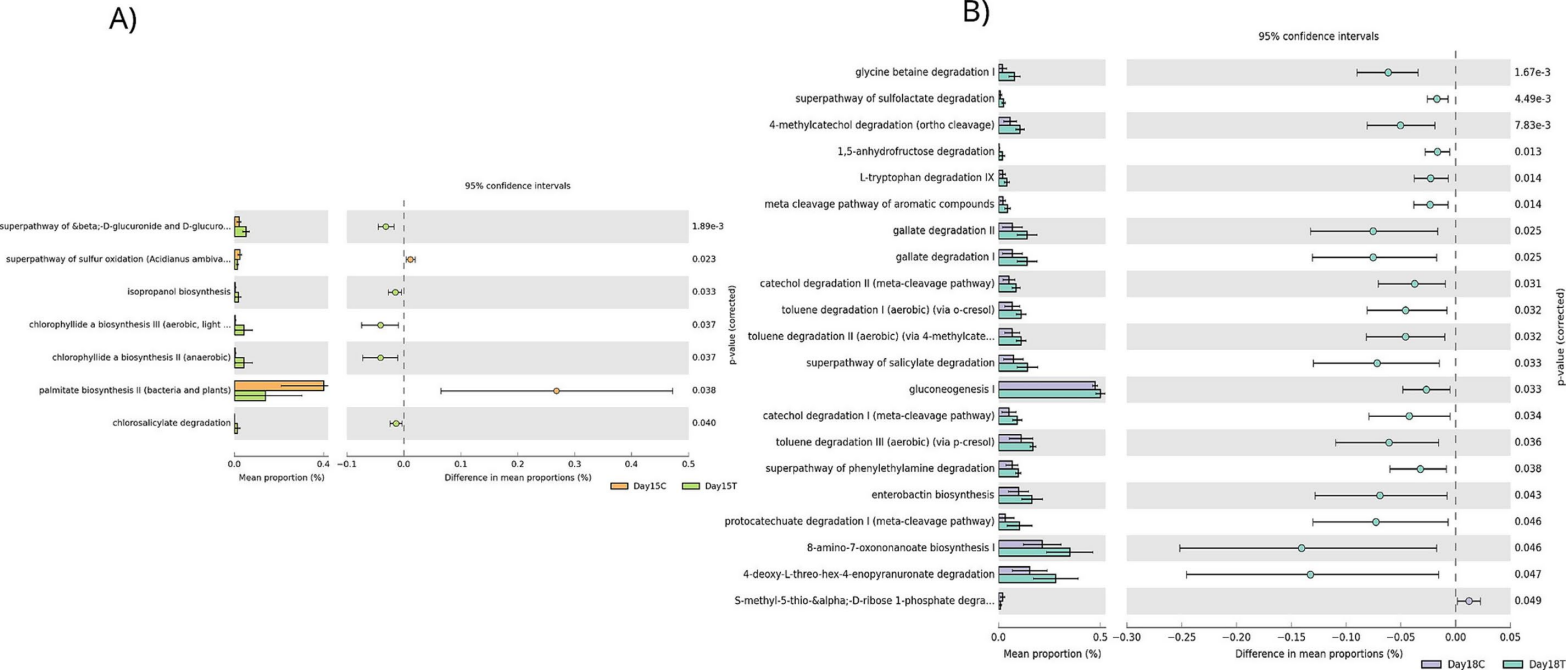
Effects of TM on microbial metabolic pathways in the CAM **(A)** Day 15C vs Day 15T **(B)** Day 18C vs Day 18T. Differential pathway abundances are shown, with effect sizes displayed on the right side of the plot as log-transformed fold changes. Points represent mean effect sizes, and horizontal lines indicate 95% confidence intervals. The effects of treatments were significantly different at *p* < 0.05 between the treatment groups.

Superpathway of beta D-glucuronide and D-glucuronate degradation, Isopropanol biosynthesis, Palmitate biosynthesis II, and chlorosalicylate degradation pathways are significantly increased (*p* < 0.05) in D15TM compared to D15C (Figure 5A).

This study showed 21 metabolic pathways in the D18C and D18TM groups (Figure 5B). However, among all these pathways, the S-methyl-5-thio-alpha-D-ribose 1-phosphate degradation pathway was significantly increased (*p* < 0.05) in D18C compared to D18TM, and other pathways were significantly decreased (*p* > 0.05).

### 3.4 CAM gene expression

**Figure 6:**
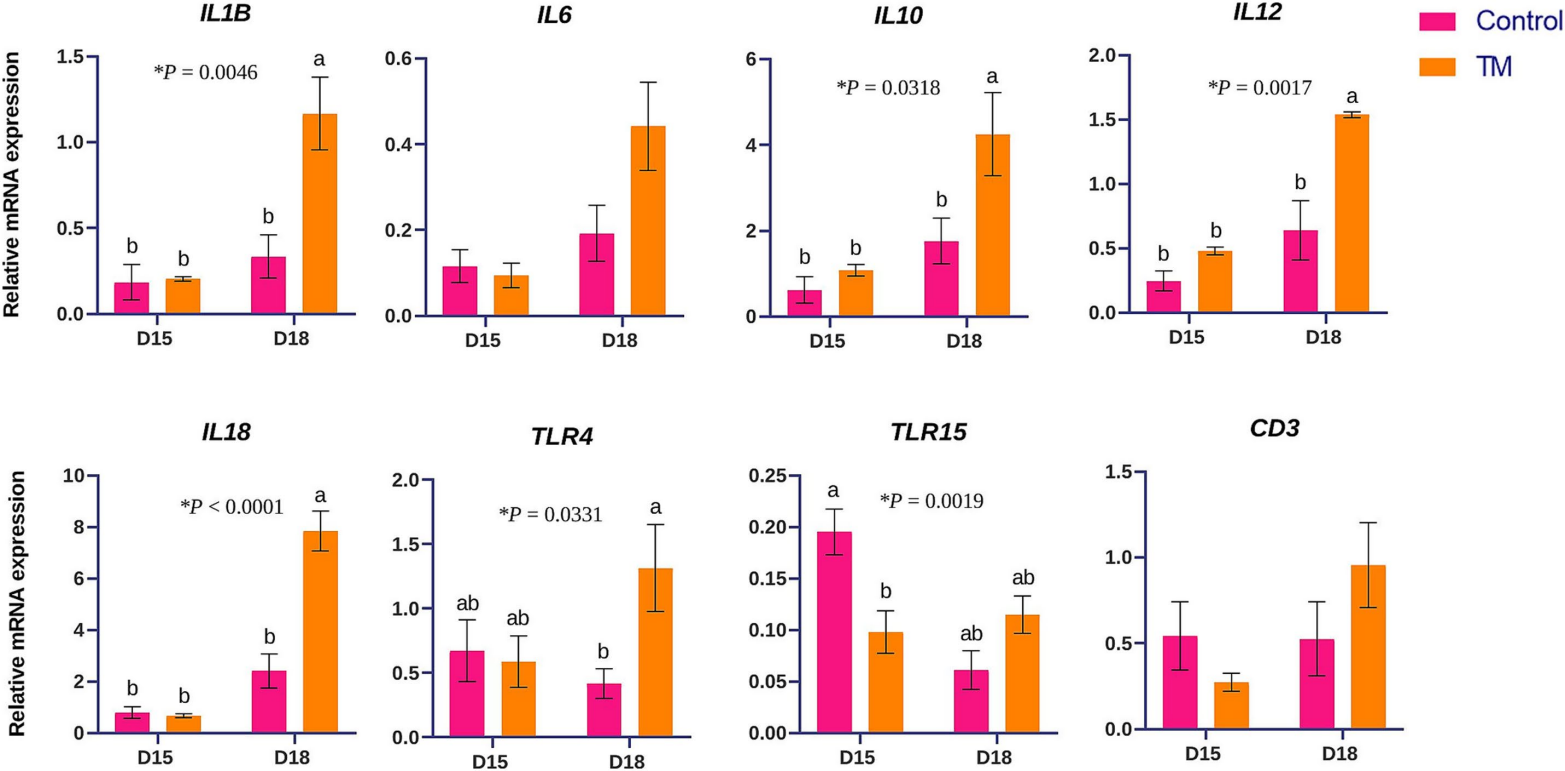
Effects of TM on the mRNA expression of immune-related genes in the CAM. Data are shown as mean ± SEM. Different letters indicate a significant difference among the treatment groups.

The expression pattern of the immune-related genes (*IL1B, IL6, IL10, IL12, IL18, TLR4, TLT15,* and *CD3*) among the treatments is summarized in Figure 6. The mRNA expressions of *IL1B* (*p* = 0.0046), *IL10* (*p* = 0.0318), *IL12* (*p* = 0.0017), and *IL18* (*p* < 0.0001) were significantly higher in the D18TM compared to the other treatment groups. *TLR4* expression was significantly higher (*p* = 0.0331) in the D18TM than in D18C. *TLR15* expression was significantly lower (*p* = 0.0019) in the D15TM compared to the D15C group.

### 3.5 Intestine microbiota profile

**Figure 7:**
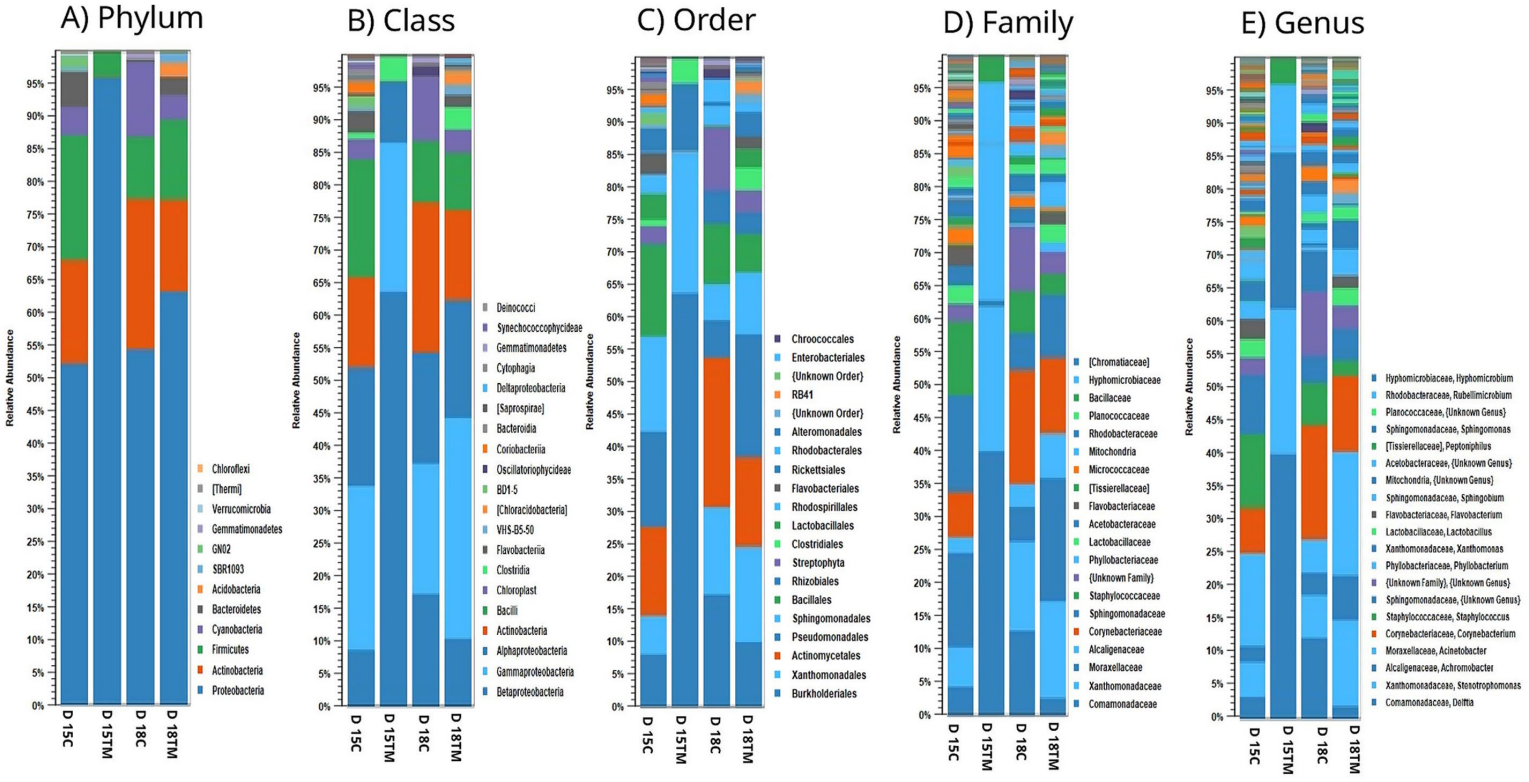
Effects of TM on the average relative abundance of the intestinal microbiota at the phylum **(A)**, class **(B)**, Order **(C)**, Family **(D)**, and Genus levels **(E)**. Data represents relative abundance (%).

This study investigated intestinal microbial profiling at the phylum, class, order, family, and genus levels. The diversity of the intestinal microbiota at the phylum level among treatment groups is shown following the exclusion of low-abundance OTUs (Figure 7A). *Proteobacteria* and *Actinobacteria* were the two most dominating phyla among the treatments in D15C (52 and 16%, respectively), D15TM (95 and 4%, respectively), D18C (54 and 22%, respectively), and D18TM (63 and 14%, respectively).

At the class level (Figure 7B), the most prevalent microbiota were *Betaproteobacteria* and *Gammaproteobacteria*. They were present in D15C (9 and 25%, respectively), D15TM (63 and 23%, respectively), D18C (17 and 20%, respectively), and D18TM (11 and 34%, respectively).

At the order level (Figure 7C), the most dominant microbiota were *Burkholderiales, Xanthomonadales,* and *Actinomycetes*. They were present in D15C (8, 6 and 14%, respectively), D15TM (64, 21 and 0.2%, respectively), D18C (17, 14 and 23%, respectively), and D18TM (10, 15 and 19% respectively).

At the family level (Figure 7D), the most dominant microbiota were *Comamonadaceae, Xanthomonadaceae,* and *Moraxellaceae*. They were present in D15C (4, 6, and 15%, respectively), D15TM (40, 22, and 1%, respectively), D18C (12, 15, and 5%, respectively), and D18TM (3, 14 and 19%, respectively).

At the genus level (Figure 7E), the most dominant microbiota were *Delftia, Stenotrophomonas*, *Achromobacter,* and *Acinetobacter.* They were present in D15C (3, 6, 2, and 14%, respectively), D15TM (40, 22, 23, and 1%, respectively), D18C (12, 7, 3 and 5%, respectively), and D18TM (2, 14, 7 and 19%, respectively).

### 3.6 Alpha and beta diversity of intestine microbiota

**Figure 8:**
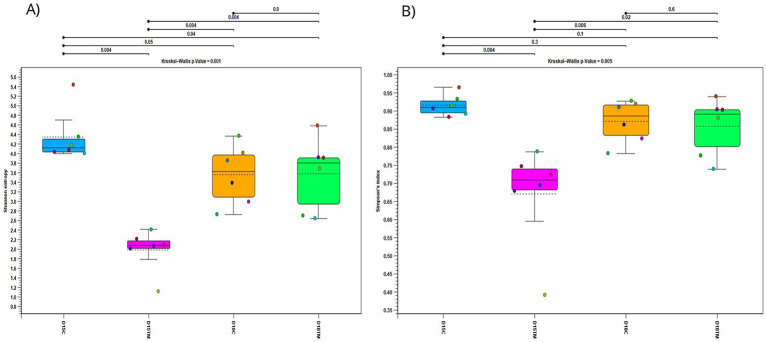
Effects of TM on microbial alpha diversity in the intestine. **(A)** Shannon entropy. **(B)** Simpson’s index. The effects of treatments were significantly different at *p* < 0.05 among the treatment groups.

This study employed Shannon entropy and Simpson’s index to assess Alpha diversity (Figure 8). In this study, Shannon entropy showed a significant difference (*p* < 0.05) in the D15C compared to the other treatment groups. Also, D15TM showed a significant difference (*p* < 0.05) from D18C and D18TM. Simpson’s index was significantly different (*p* < 0.05) in the D15C compared to D15TM. The D15TM was significantly different (*p* < 0.05) compared to D18C and D18TM.

**Figure 9:**
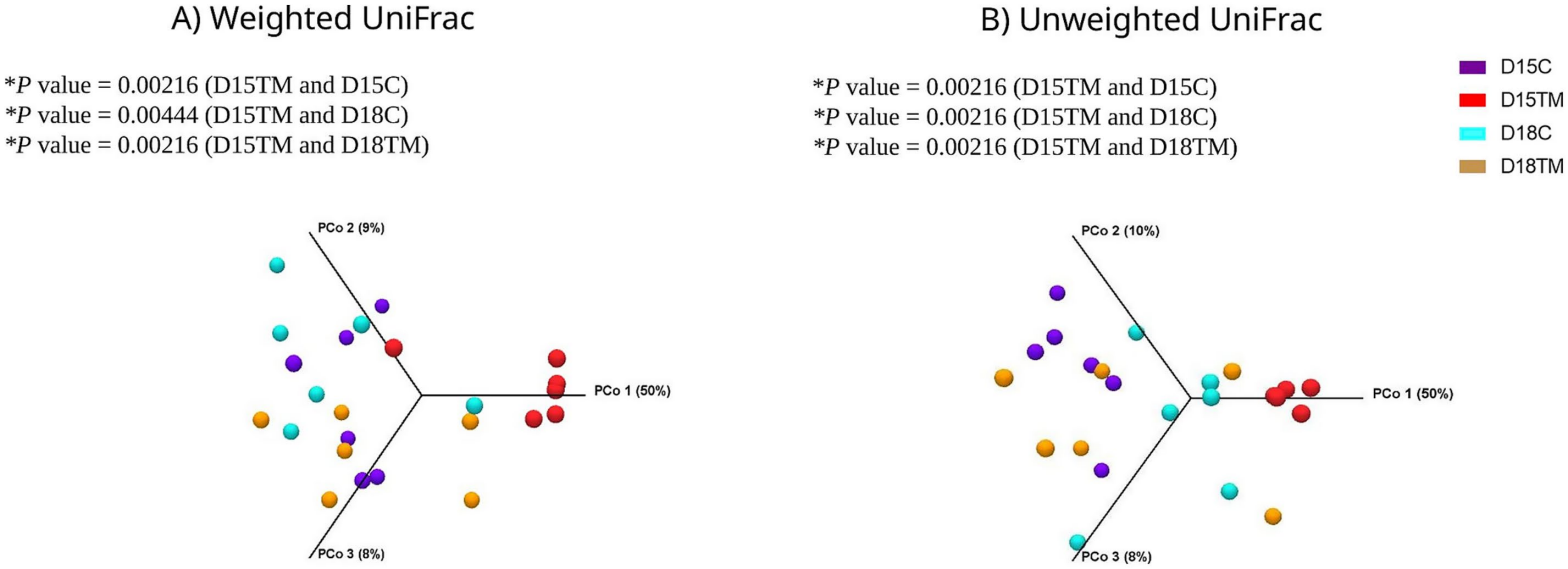
Effects of TM on microbial beta diversity in the intestine. **(A)** Weighted UniFrac; **(B)** Unweighted UniFrac. The effects of treatments were significantly different at *p* < 0.05 among the treatment groups.

This study assessed Beta diversity through weighted UniFrac and unweighted UniFrac (Figure 9). The weighted UniFrac shows a significant difference in the D15TM group compared to the D15C, D18C, and D18TM (*p* = 0.00216, *p* = 0.00444, and *p* = 0.00216, respectively). The Unweighted UniFrac shows a significant difference in the D15TM group compared to the D15C, D18C, and D18TM (*p* = 0.00216, *p* = 0.00216, and *p* = 0.00216, respectively). The D15C group differs significantly from the D18C and D18TM groups (*p* = 0.00216 and *p* = 0.03247, respectively).

### 3.7 Intestine microbial metabolic pathways

**Figure 10:**
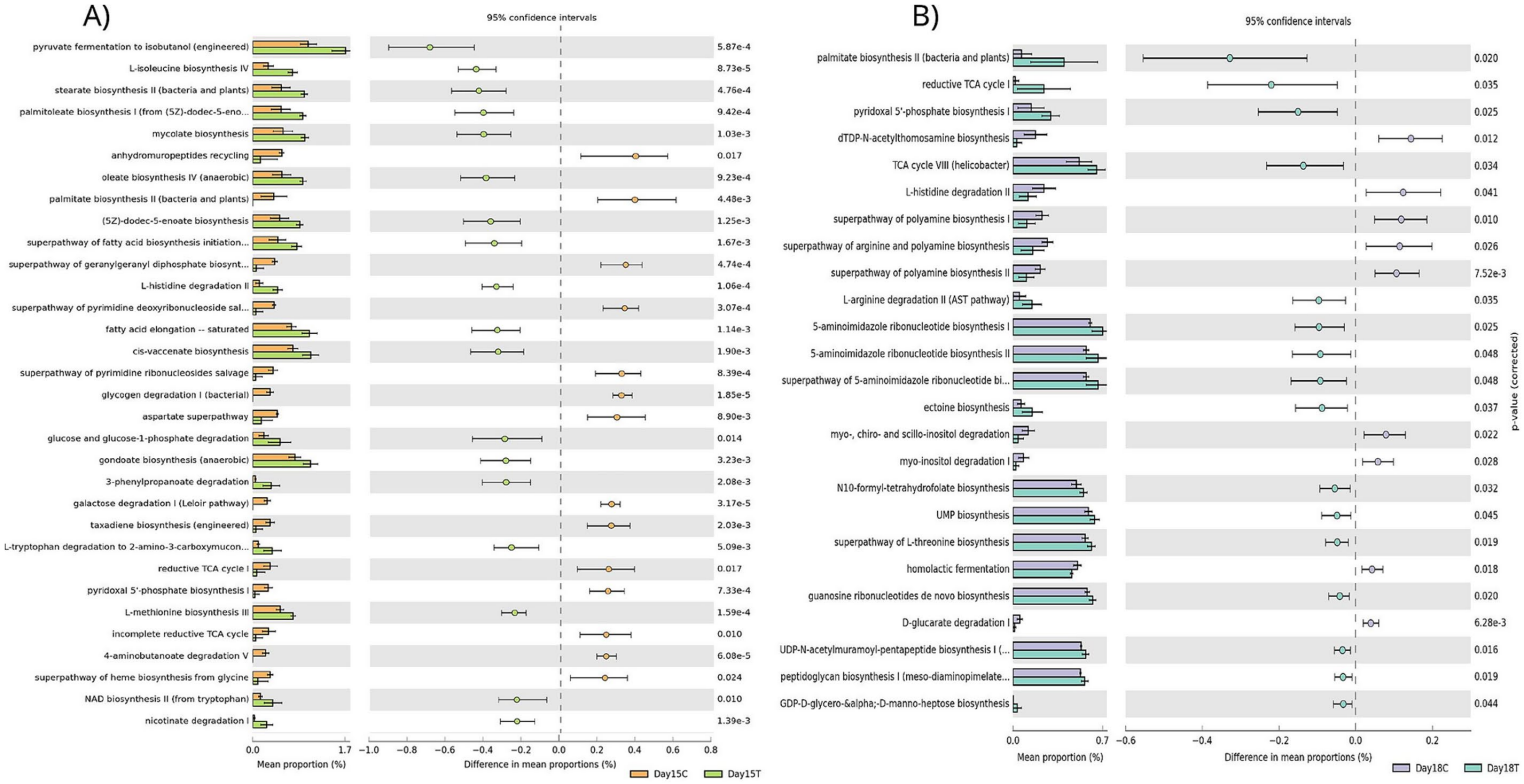
Effects of TM on microbial metabolic pathways in the intestine **(A)** Day 15C vs Day 15T **(B)** Day 18C vs Day 18T. Differential pathway abundances are shown, with effect sizes displayed on the right side of the plot as log-transformed fold changes. Points represent mean effect sizes, and horizontal lines indicate 95% confidence intervals. The effects of treatments were significantly different at *p* < 0.05 between the treatment groups.

This study showed 21 metabolic pathways in the D15C and D15TM groups (Figure 10A). Among them, notably pyruvate fermentation to isobutanol (engineered), L-isoleucine biosynthesis IV, and mycolate biosynthesis pathways were significantly increased (*p* < 0.05) in D15TM compared to D15C.

We also analyzed 25 metabolic pathways in the D18C and D18TM groups (Figure 10B). Among them, reductive TCA cycle I, Pyridoxal 5′-phosphate biosynthesis I, 5-aminoimidazole ribonucleotide biosynthesis I and II pathways were significantly increased (*p* < 0.05) in D18TM compared to D18C.

### 3.8 Intestinal gene expression

**Figure 11:**
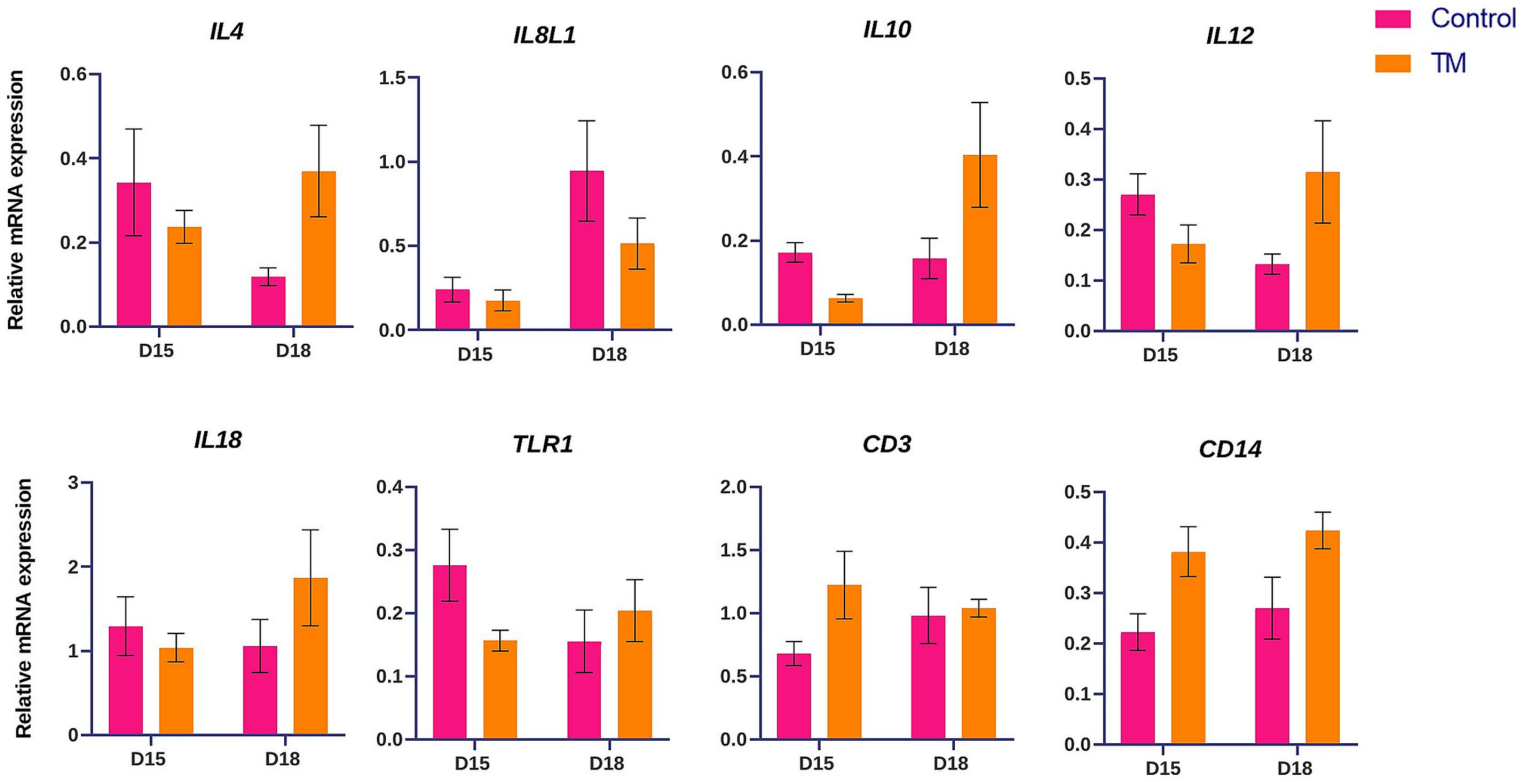
Effects of TM on the mRNA expression of immune-related genes in the intestine. Data are shown as mean ± SEM. Different letters indicate a significant difference between the groups.

The expression patterns of the immune-related genes (*IL4*, *IL8L1, IL10, IL12, IL18, TLR1, CD3,* and *CD14*) among the treatments are summarized in Figure 11. There was no significant difference (*p* < 0.05) among the treatments in these gene expressions.

### 3.9 Specific bacterial abundance in CAM and intestinal microbiota

**Figure 12:**
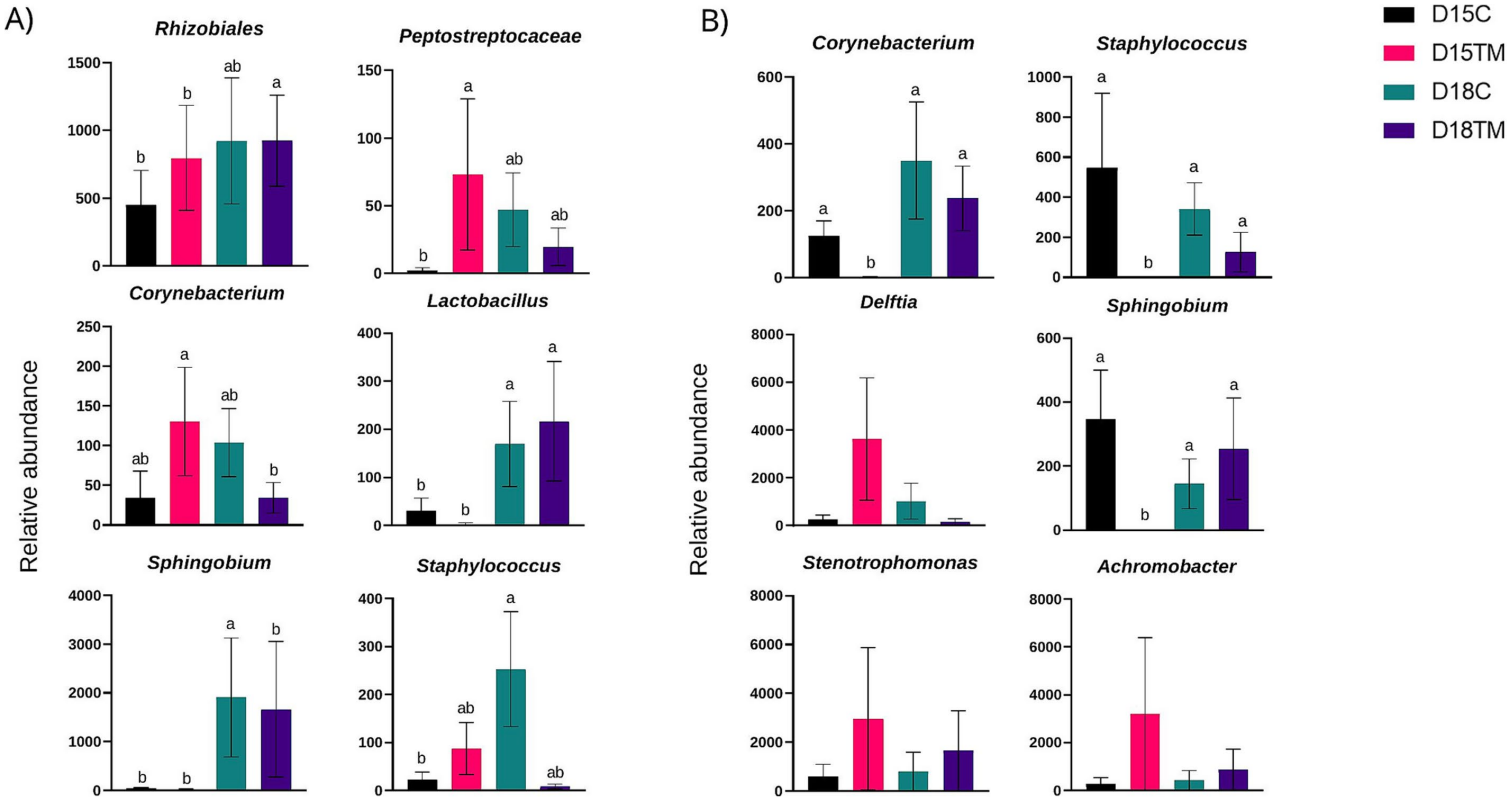
Effects of TM on the significantly abundant microbiota at the order, family, and genus level in the CAM **(A)** and intestine **(B)**. Data are shown as mean ± SEM. Different letters indicate a significant difference among the treatment groups.

Specific bacterial abundances of the CAM microbiota at the order, family, and genus levels are presented in Figure 12A. At the order level, *Rizobiales* were significantly increased (*p* < 0.05) in D18TM compared to D15C and D15TM. At the family level, *Peptostreptococaceae* were significantly increased (*p* < 0.05) in D15TM compared to D15C. At the genus level, *Corynebacterium* was significantly increased (*p* < 0.05) in D15TM compared to D18TM. *Lactobacillus* and *Sphingobium* significantly increased (*p* < 0.05) in D18C and D18TM compared to D15C and D15TM.

Specific bacterial abundance of intestine microbiota at the genus level is presented in Figure 12B. At the genus level, *Corynebacterium, Staphylococcus*, and *Sphingobium* significantly decreased (*p* < 0.05) in D15TM compared to the other treatment groups.

### 3.10 Correlation analysis between CAM and intestine

**Figure 13:**
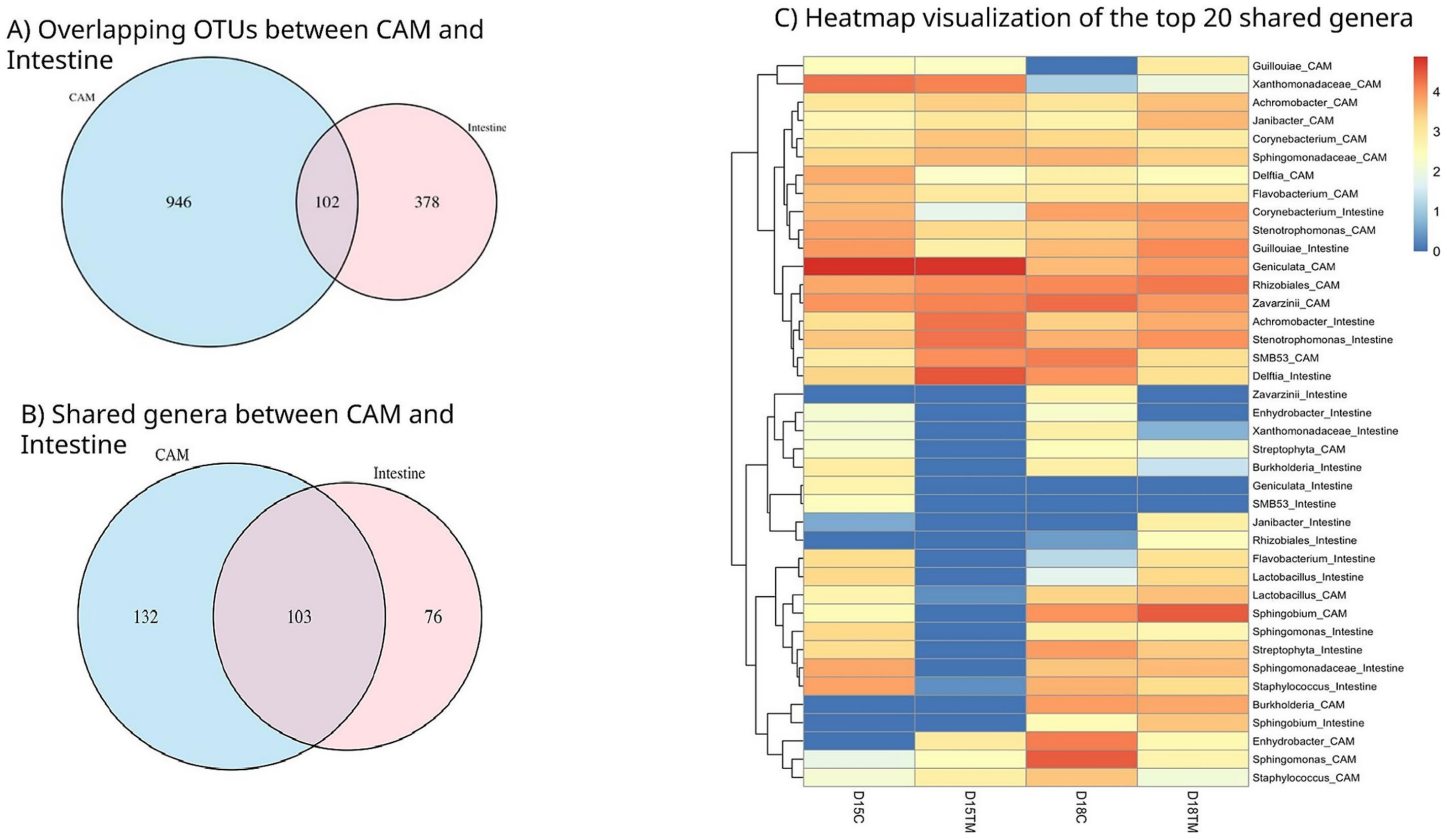
Shared microbial taxa between the CAM and Intestine. **(A)** Venn diagram showing overlapping OTUs detected in CAM and intestinal samples. **(B)** Venn diagram showing the number of bacterial genera shared between the two tissues. **(C)** Heatmap illustrating the relative abundance of selected shared genera across experimental groups. Colors represent log-transformed relative abundance, with blue indicating low abundance and red indicating higher abundance.

To examine microbial overlap between embryonic tissues, we compared microbial communities detected in CAM and intestinal samples. As shown in Figures 13A, a total of 102 OTUs were shared between CAM and intestinal samples, whereas 946 OTUs were unique to CAM and 378 OTUs were unique to intestinal samples. Similarly, analysis at the genus level revealed 103 shared genera between the two tissues, with 132 genera unique to CAM and 76 unique to the intestine (Figure 13B).

To further evaluate the distribution of shared taxa, a heatmap was generated to visualize the relative abundance patterns of the top 20 most abundant shared genera across experimental groups (Figure 13C). A detailed correlation analysis of all the available taxa is shown in Supplementary Table 2. The heatmap shows that several bacterial genera were detected in both tissues across developmental stages and treatment groups, including *Sphingomonas*, *Staphylococcus*, *Burkholderia*, *Sphingobium*, and *Flavobacterium*. The abundance patterns of these shared taxa varied across embryonic stages (ED15 vs. ED18) and treatments (control vs. TM). In general, several taxa exhibited higher relative abundance at ED18 compared with ED15, and some genera displayed differences between control and TM groups.

## 4 Discussion

### 4.1 CAM microbiome characterization

As of today, there is no reported evidence of CAM microbiota composition. Our study showed that, at the phylum, class, order, family, and genus levels, microbial diversity was lower in the D15 groups than in the D18 groups. The differences in microbial diversity at various embryonic stages may be attributed to the fact that ED15 is considered to be in the mid-embryonic phase (Hamburger and Hamilton, 1992). During this mid-embryonic phase, the microbiome is less diverse, whereas greater microbial diversity is observed at the late embryonic stage, such as ED18. Notably, the D15TM group exhibited a more balanced microbial diversity compared to the D15C group. At this stage, the embryo grows rapidly and can adapt to fluctuations in incubation temperature (Tzschentke, 2008). Additionally, around ED15, embryos begin to respond to auditory stimuli and develop frequency selectivity (Jones et al., 2006). D18TM exhibited a more balanced bacterial population at both the family and genus levels compared to D18C. This indicates that embryonic TM can influence microbial diversity in CAM. We have identified the genus *Phenylobacterium*, which is important for decomposing xenobiotic compounds, such as the herbicide chloridazon. It also plays a role in bioremediation, using bacteria and fungi to break down environmental pollutants into less harmful substances (Li et al., 2019). *Phenylobacterium* has been described in environmental contexts and was detected in CAM in the present study; however, its functional role in avian embryos remains unknown*. Limnobacter* is another type of bacteria that participates in sulfur oxidation, oxidative phosphorylation, and ethanol fermentation, performing both aerobic and anaerobic functions. It may contribute to the anaerobic methane-oxidizing community by supplying sulfate derived from sulfur oxidation (Chen et al., 2016). Additionally, it may facilitate gas exchange through the Crassulacean Acid Metabolism process.

### 4.2 Alpha and beta diversity in CAM

Alpha diversity quantifies microbiota diversity within the treatments (Al Amaz et al., 2024a). This study utilized Shannon entropy and Simpson’s index to assess alpha diversity in CAM. Shannon entropy measures species richness by quantifying uncertainty in species identification, while Simpson’s index indicates the probability that two randomly selected individuals belong to different species, reflecting their relative abundance (Chaudhary et al., 2023). Both measurements showed significantly higher microbial abundance in the D18 group than in the D15 group. Beta diversity assesses the variation in microbial composition across different treatment groups. Using both weighted and unweighted UniFrac analyses, significant differences were observed between the D15TM group and the D15C, D18C, and D18TM groups. Additionally, the D15C group differed significantly from the D18C and D18TM groups. These results indicate that bacterial abundance varies significantly between the D15 and D18 treatment groups. Notably, the microbial diversity between the D15C and D15TM groups suggests that embryonic treatment can significantly impact microbial composition in broiler embryos.

### 4.3 Predicated metabolic pathways in CAM

This study explored microbial metabolic pathways in the D15 and D18 treatment groups in CAM. In the D15TM group, metabolic pathways were significantly more active than in D15C, particularly those involved in isopropanol biosynthesis. This process converts acetyl-CoA to acetone, which is then reduced to isopropanol (Shi et al., 2022). In the D18TM group, nearly all pathways showed significantly higher levels than in the D18C group, except for the S-methyl-5-thio-alpha-D-ribose 1-phosphate degradation pathway. Specifically, the glycine betaine degradation I and gluconeogenesis I pathways were notably elevated in D18TM when compared to D18C. Glycine is a key amino acid in both mammals and chickens, but avian species typically do not synthesize enough glycine internally. The glycine betaine degradation I pathway produces ammonia and carbon dioxide in animals (Wang et al., 2013), however, its direct contribution to embryonic gaseous exchange through CAM remains speculative and requires validation using metagenomic or metabolomic approaches. Gluconeogenesis is a metabolic pathway that produces glucose from non-carbohydrate sources, such as lactate, glycerol, and specific amino acids (Wang et al., 2024). In this context, the predicted enrichment of gluconeogenesis-related pathways may suggest that CAM actively supplies glucose to the embryo, enhancing metabolism and growth rates. This aligns with our previous finding (Amaz et al., 2024) that higher embryonic metabolism is associated with shorter hatching times. Overall, embryonic TM significantly impacts microbial metabolic pathways in CAM, aiding embryo growth.

### 4.4 Gene expressions in CAM

In this study, we also examined key immune-related genes in the CAM, including *IL1B, IL10, IL12, IL18, TLR4,* and *TLR15*, which showed significant differences among treatments. IL1B is vital for inflammatory responses and autoinflammatory disorders (He et al., 2011). IL10, known for its anti-inflammatory effects, helps limit immune responses to pathogens, protecting the host and maintaining tissue homeostasis. Dysregulation of IL10 is associated with increased immunopathology and susceptibility to autoimmune diseases. The IL12 gene encodes the cytokine IL-12, which is crucial for regulating cell-mediated immunity against intracellular pathogens by stimulating natural killer (NK) cells and T-helper 1 (Th1) cells to produce interferon-gamma (Su et al., 2011). IL18 also plays a role in immune responses, activating Th1 cells, macrophages, NK cells, natural killer T (NKT) cells, B cells, dendritic cells (DCs), and non-polarized T cells to generate interferon-gamma (IFN-*γ*) alongside IL-12 (Ihim et al., 2022). Toll-like receptor 4 (TLR4) detects bacterial lipopolysaccharides and other pathogen components, triggering the production of pro-inflammatory cytokines and initiating both immediate and prolonged adaptive immune responses (Vaure and Liu, 2014). In our study, genes were significantly upregulated in the D18TM group compared to the D18C group, indicating that TM influences cell-mediated CAM immunity and has a lasting effect on post-hatch immunity (Amaz et al., 2025a). TLR15, an avian-specific receptor activated by yeast lysates, contributes to the innate immune response against fungal infections (Ciraci and Lamont, 2011). However, *TLR15* expression was lower in the D15TM group, likely because embryos were still adapting to elevated temperatures, leading to a reduced immune response.

### 4.5 Intestine microbiome characterization

Previous studies on embryonic intestinal microbiota are limited. We assessed microbial diversity at various taxonomic levels and found that the D15TM group had lower diversity than the other treatment groups. In contrast, D18TM showed greater diversity than D18C. This suggests that the TM treatment notably affects microbial diversity, and exposure to elevated temperatures results in increased diversity by D18TM. *Delftia*, an opportunistic pathogenic bacterium, was identified as having lower abundance in D18TM than in D18C. It was detected in antibiotic-treated biting midges but was scarce in antibiotic-treated mosquitoes (Möhlmann et al., 2020). *Phyllobacterium* are bacteria found on tree leaf surfaces that can affect host plants in beneficial, harmful, or neutral ways, including promoting growth and influencing nutrient cycling (Coutinho and Bophela, 2021). *Flavobacterium* also supports plant health by enhancing growth, managing diseases, and increasing resilience to abiotic stress (Seo et al., 2024). Both genera are commonly described in plant-associated environments. Their detection in embryonic intestine suggests possible environmental or transient colonization; however, their role in avian development remains to be determined. *Rubelimicrobium,* a member of the Roseobacter group, is important to marine biogeochemical cycles and has been discovered in environments linked to algal blooms (Riedel et al., 2014). It may also play a role in intestinal biochemical pathways. More research is needed to clarify the roles of these bacteria in the embryonic intestine. Furthermore, genera such as *Achromobacter, Stenotrophomonas, Lactobacillus, and Staphylococcus* are commonly found in intestines and the conceptus of avian embryos, suggesting an overlapping microbiota between the CAM and intestine in broiler embryos.

### 4.6 Alpha and beta diversity in the intestine

Alpha diversity (Shannon entropy and Simpson’s index) and beta diversity (Weighted and Unweighted UniFrac) significantly differed between the D15TM and D15C groups in the intestine, while no differences were found in the D18TM and D18C groups. This may be due to thermal management starting at ED12; at ED15, embryos were not yet acclimatized to the elevated temperature. By ED18, they had adapted, leading to a more balanced microbial composition.

### 4.7 Predicted metabolic pathways in the intestine

The study identified microbial metabolic pathways in the D15 and D18 treatment groups. In D15TM, 18 metabolic pathways were significantly increased compared to D15C, notably L-isoleucine biosynthesis IV, mycolate biosynthesis, and L-histidine degradation II. The L-isoleucine biosynthesis IV pathway consists of five steps, with the last four steps identical to those in the valine biosynthesis pathway, highlighting the interconnected superpathway of branched-chain amino acid biosynthesis (Cotton et al., 2020). The mycolate biosynthesis pathway is essential for the cell wall structure of mycobacteria, involving the production of mycolic acids through a fatty acid synthase type II (FAS-II) system and a condensation mechanism (Bailo et al., 2022). Meanwhile, L-histidine degradation II is a metabolic pathway that breaks down histidine into glutamate and other metabolites, starting with the conversion of histidine to urocanic acid (Hug et al., 1999). Microbial metabolic pathways in D18TM were significantly higher than in D18C, particularly in palmitate biosynthesis II, the reductive TCA cycle I, L-histidine degradation II, and the 5-aminoimidazole ribonucleotide pathways. In chickens, palmitate biosynthesis occurs mainly in the liver through acetyl-CoA condensation, aided by the fatty acid synthase (FAS) enzyme (Anderson and Hammes, 1984). The reductive TCA cycle I functions in reverse, fixing two CO_2_ molecules and producing one acetyl-CoA, which is converted to pyruvate, a precursor for other metabolites (Hügler et al., 2005). The 5-aminoimidazole ribonucleotide I and II pathway is a key intermediate in purine nucleotide and thiamine biosynthesis (Groziak et al., 1988). These predicted pathways may enhance microbial metabolism, potentially improving embryonic gut health and nutrient absorption, with lasting benefits for post-hatch growth performance as found in our previous study (Al Amaz et al., 2024a). However, functional validation is required to conclusively determine the effects of these pathways on embryonic growth, as direct evidence of enhanced metabolism or nutrient absorption was not assessed in the present study.

### 4.8 Gene expression in the intestine

We analyzed the expression of several immune-related genes in the Intestine, including *IL4, IL8L1, IL10, IL12, IL18, TLR1, CD3,* and *CD14*. However, we found no significant differences between the D15 and D18 treatment groups. A previous study (Eren et al., 2016) indicates that while immune development begins at ED3, innate immunity typically matures by ED18, as gut immunity matures within 5 to 10 days post-hatch (Song et al., 2021). The lack of substantial changes in intestinal immune gene expression should not be considered just as indication of inadequate immune development. Immune development during embryogenesis is presumably tissue-specific, with the CAM and gut potentially facing different immunological stresses. The CAM is a highly vascularized extraembryonic membrane that interacts with the eggshell and the external environment, possibly subjecting it to earlier or distinct immunological responses compared to the growing intestinal mucosa. Conversely, intestinal immune modulation during late embryogenesis may be influenced by advancing structural maturity and by specific signaling mechanisms distinct from those of the CAM. Furthermore, nuanced transcriptional variations in the intestine may have gone undetected due to the use of only gene expression data. The absence of notable intestinal immune alterations likely indicates tissue-specific developmental dynamics rather than a lack of immune response.

### 4.9 Significantly abundant microbiota in CAM and intestine

We performed a detailed analysis of the significant microbiota abundance at the order, family, and genus levels to further investigate the CAM microbial community. At the order level, we found that *Rhizobiales* were significantly more abundant in the D18TM group compared to the D15 group. *Rhizobiales* play a beneficial role in plant-microbe interactions and can form nitrogen-fixing symbiotic associations with leguminous plants (Rosselli et al., 2021). At the family level, *Peptostreptococcaceae* was significantly higher in the D15TM group than in the D15C group. *Peptostreptococcaceae* are the standard commensals of the gastrointestinal tract, which maintain gut homeostasis (Al Amaz et al., 2024a). *Sphingobium* was significantly higher in the D18 group than in the D15 group. *Sphingibium* primarily comprises various environmental isolates that contribute to bioremediation and the biodegradation of pollutants (Boss et al., 2022). Its presence in the embryonic tissues in the current study could be due to a temporary environmental exposure. *Staphylococcus* was significantly higher in D18C compared to D15C, but lower in the D18TM group. While commonly present in healthy poultry, *Staphylococcus* can induce localized or systemic infections if skin integrity or mucous membrane barriers are breached (Syed et al., 2020). Taken together, TM significantly influenced the composition of the beneficial bacterial population in CAM.

We further analyzed significant microbiota abundance at the genus level to examine the intestinal microbial community. *Corynebacterium, Staphylococcus,* and *Sphingobium* were significantly decreased in D15TM than in the other treatment groups. *Corynebacterium* may contribute to pathogen defense, potentially decreasing vulnerability to specific infections (Memon et al., 2022). *Staphylococcus and Sphingobium* have already been discussed above. Intestinal microbial diversity was significantly impacted by the D15TM treatment, likely due to the increased temperature. While the embryos were still adjusting at ED15, by ED18, they had acclimated, with no significant differences between the treatment groups.

### 4.10 Potential evidence of vertical transmission

The analysis of microbial communities in the CAM and intestine demonstrated significant overlaps in microbial taxa, suggesting possible microbial transfer during embryonic growth and development. A total of 102 overlapping OTUs, whereas 103 bacterial genera were shared in both CAM and intestinal samples. This overlap suggests that specific microbial taxa may be present in both the CAM and the intestine. The heatmap analysis further delineates the abundance patterns of selected genera identified in both CAM and intestinal samples across developmental stages and treatment groups. Genera including *Sphingomonas, Staphylococcus, Burkholderia*, and *Sphingobium* were identified in both tissues, exhibiting higher relative abundance at ED18 than at ED15, suggesting potential microbial alterations during embryonic development. Furthermore, specific genera exhibited identical responses to TM, especially at ED18, when taxa such as *Lactobacillus* and *Sphingobium* were more prevalent in the TM group relative to the controls. The clustering pattern also aggregated numerous CAM-associated taxa alongside their intestinal equivalents, indicating similarities in abundance dynamics across tissues. These data indicate that CAM and gut microbial communities may possess shared taxa and developmental trajectories during late embryogenesis.

While the observed overlap between CAM and the intestinal microbiota is consistent with vertical transmission, alternative explanations cannot be ruled out. The shared taxa could arise from (i) common environmental exposure during sample collection, (ii) bidirectional transfer, or (iii) independent colonization from egg contents. Many of the overlapping genera (*Sphingomonas, Staphylococcus, Burkholderia, Sphingobium*) are ubiquitous environmental organisms and common laboratory contaminants, necessitating cautious interpretation. Future studies using strain-level identification and temporal sampling to show CAM colonization before intestinal colonization, along with stringent contamination controls, are needed to clearly define vertical transmission pathways.

Although this study offers novel insights into the microbial composition of the CAM and its possible influence on embryonic intestinal development, it has limitations. A major limitation of the present study is the lack of thorough environmental contamination controls. There was no mock or positive community included. Embryonic tissues constitute low-biomass samples and are more vulnerable to background contamination from incubator conditions, eggshell surfaces, laboratory air, chemicals, or extraction kits. Despite the implementation of stringent aseptic protocols, controls for the DNA extraction kit, environmental swabs (from incubator surfaces and eggshells), air samples, and water blanks were not processed concurrently with tissue samples. This constraint necessitates careful interpretation of microbial composition, especially for taxa frequently documented in environmental contexts. Further studies integrating SPF eggs and thorough contamination controls will be crucial to substantiate these findings. The total microbial load was not quantified using qPCR of 16S rRNA gene copies. Thus, the observed differences reflect relative microbial composition and predicted functional profiles, rather than absolute bacterial abundance. It is important to note that PICRUSt2 provides predictions of functional potential based on reference genomes and does not directly measure gene abundance, gene expression, or metabolic activity. Therefore, inferred pathways represent predicted functional capacity rather than experimentally validated function. Using larger sample sizes would further strengthen the statistical analysis and increase the ability to detect smaller biological effects. Additionally, some shared genera between CAM and the intestine may indicate environmental or technical overlaps instead of actual biological transmission. This is particularly relevant considering the low biomass of CAM samples and the limited strain-level resolution provided by 16S rRNA profiling.

## 5 Conclusion

To the best of our knowledge, this study is the first to determine the microbial composition of CAM. Our findings identified the presence of *Phenylobacterium* and *Limnobacter* in CAM, which may contribute to detoxification and gaseous exchange. Additionally, TM significantly increased both the microbial diversity and metabolic pathways in CAM and the intestines. Moreover, TM notably enhanced the expression of immune genes in CAM. In the intestinal environment, we discovered *Phyllobacterium, Flavobacterium,* and *Rubelimicrobium*, which may support growth, reduce stress, and improve nutrient absorption and biochemical pathways. We found that *Achromobacter, Stenotrophomonas, Lactobacillus,* and *Staphylococcus* exhibited the highest relative abundance at the genus level in both the CAM and intestine. This suggests vertical transmission of microbiomes from the CAM to the intestine, though the process appeared somewhat limited.

## Data Availability

The raw data generated in this study can be found in the NCBI BioProject repository under accession PRJNA1263685.
