# Supplementary material for: Limited vertical transmission of microbiomes through the chorioallantoic membrane affects intestine development and metabolic pathways in broiler embryos
Source: Front Microbiol. 2026 Jul 13;17:1780310. doi: 10.3389/fmicb.2026.1780310 (PMC13402465; doi:10.3389/fmicb.2026.1780310)
Supplement: Supplementary file 2 [file Table_2.DOCX]

**Supplement Table 2:** Values are reported as Spearman correlation coefficients (rho) with corresponding p-values for CAM and Intestine microbiota.

| **Genus** | **Spearman rho** | **p-value** |
| --- | --- | --- |
| *Bacillales* | 1 | 0 |
| *Sediminibacterium* | 1 | 0 |
| *Nitroreducens* | 1 | 0 |
| *MLE1-12* | 1 | 0 |
| *Pseudoxanthomonas* | 1 | 0 |
| *Aminobacter* | 1 | 0 |
| *Rhizobiales* | 0.949 | 0.05132 |
| *Sphingobium* | 0.949 | 0.05132 |
| *Psychrophilus* | 0.949 | 0.05132 |
| *BD1-5* | 0.949 | 0.05132 |
| *Brevibacterium* | -0.949 | 0.05132 |
| *Mesophilicum* | -0.949 | 0.05132 |
| *Betaproteobacteria* | -0.943 | 0.05719 |
| *Hypermegale* | 0.816 | 0.1835 |
| *Cloacibacterium* | 0.816 | 0.1835 |
| *Cryptophyta* | 0.778 | 0.2222 |
| *Rhodospirillaceae* | 0.778 | 0.2222 |
| *Conglomeratum* | -0.775 | 0.2254 |
| *Clostridiaceae* | -0.775 | 0.2254 |
| *Microbacteriaceae* | -0.775 | 0.2254 |
| *SMB53* | -0.775 | 0.2254 |
| *Geodermatophilaceae* | -0.775 | 0.2254 |
| *Sphingopyxis* | -0.775 | 0.2254 |
| *Geniculata* | 0.775 | 0.2254 |
| *Myxococcales* | 0.775 | 0.2254 |
| *Bradyrhizobiaceae* | 0.775 | 0.2254 |
| *[Ruminococcus]* | 0.775 | 0.2254 |
| *Zavarzinii* | 0.775 | 0.2254 |
| *Aureum* | 0.775 | 0.2254 |
| *Yanoikuyae* | 0.738 | 0.2621 |
| *Actinomycetales* | 0.738 | 0.2621 |
| *Lactobacillus* | 0.8 | 0.3333 |
| *Micrococcaceae* | 0.8 | 0.3333 |
| *Stenotrophomonas* | -0.8 | 0.3333 |
| *Enterobacteriaceae* | -0.8 | 0.3333 |
| *Achromobacter* | 0.8 | 0.3333 |
| *Sphingomonadaceae* | -0.8 | 0.3333 |
| *Streptophyta* | 0.8 | 0.3333 |
| *Guillouiae* | 0.8 | 0.3333 |
| *Rheinheimera* | -0.632 | 0.3675 |
| *Bartonellaceae* | -0.544 | 0.4557 |
| *Chitinophagaceae* | -0.544 | 0.4557 |
| *Candidatus Rhodoluna* | -0.544 | 0.4557 |
| *Dermabacter* | -0.544 | 0.4557 |
| *Bacillus* | -0.544 | 0.4557 |
| *Novosphingobium* | -0.544 | 0.4557 |
| *Equorum* | -0.544 | 0.4557 |
| *Rubellimicrobium* | -0.544 | 0.4557 |
| *Pseudomonadaceae* | -0.544 | 0.4557 |
| *Stationis* | 0.5 | 0.5 |
| *Leptothrix* | -0.5 | 0.5 |
| *Gemmatimonadales* | -0.389 | 0.6111 |
| *Acetobacteraceae* | -0.389 | 0.6111 |
| *Facklamia* | -0.333 | 0.6667 |
| *Oscillospira* | -0.333 | 0.6667 |
| *Calothrix* | -0.333 | 0.6667 |
| *Veillonellaceae* | -0.333 | 0.6667 |
| *Intrasporangiaceae* | -0.333 | 0.6667 |
| *Lysobacter* | -0.333 | 0.6667 |
| *Phormidiaceae* | -0.333 | 0.6667 |
| *Sphingobacteriaceae* | -0.333 | 0.6667 |
| *Janibacter* | 0.316 | 0.6838 |
| *Mesorhizobium* | 0.316 | 0.6838 |
| *Enhydrobacter* | 0.316 | 0.6838 |
| *Salinicoccus* | -0.316 | 0.6838 |
| *Bacillaceae* | -0.316 | 0.6838 |
| *Rhizobiaceae* | 0.272 | 0.7278 |
| *Prauseria* | 0.272 | 0.7278 |
| *Bacteroides* | 0.272 | 0.7278 |
| *Coprococcus* | 0.272 | 0.7278 |
| *Solibacterales* | 0.272 | 0.7278 |
| *Streptococcus* | 0.258 | 0.7418 |
| *C111* | 0.258 | 0.7418 |
| *Reuteri* | 0.258 | 0.7418 |
| *Mitochondria* | 0.258 | 0.7418 |
| *Mycobacterium* | -0.258 | 0.7418 |
| *Chryseobacterium* | -0.258 | 0.7418 |
| *Clostridiales* | -0.258 | 0.7418 |
| *Hyphomicrobium* | -0.258 | 0.7418 |
| *Ellin6075* | -0.258 | 0.7418 |
| *Xanthomonadaceae* | -0.4 | 0.75 |
| *Corynebacterium* | -0.4 | 0.75 |
| *Flavobacterium* | 0.4 | 0.75 |
| *Delftia* | -0.4 | 0.75 |
| *Oxalobacteraceae* | -0.211 | 0.7892 |
| *Rhizophila* | 0.211 | 0.7892 |
| *Acinetobacter* | -0.105 | 0.8946 |
| *Caulobacteraceae* | -0.105 | 0.8946 |
| *Burkholderia* | 0.105 | 0.8946 |
| *Sphingomonas* | -0.2 | 0.9167 |
| *Planococcaceae* | -0.2 | 0.9167 |
| *Comamonadaceae* | 0.2 | 0.9167 |
| *Ruminococcaceae* | 0.2 | 0.9167 |
| *Lactococcus* | -0.056 | 0.9444 |
| *Dorea* | -0.056 | 0.9444 |
| *Kocuria* | -0.056 | 0.9444 |
| *Deinococcus* | -0.056 | 0.9444 |
| *Coriobacteriaceae* | -0.056 | 0.9444 |
| *Staphylococcus* | 0 | 1 |
| *Pseudomonas* | 0 | 1 |
| *Mucilaginosa* | N/A | N/A |
| *Nakamurellaceae* | N/A | N/A |
| *Prevotella* | N/A | N/A |
